# Clinical characteristics, treatment patterns and relapse in patients with clinical stage IS testicular cancer

**DOI:** 10.1007/s00345-021-03889-x

**Published:** 2021-12-02

**Authors:** Maximilian Peter Brandt, C. Ruf, K. P. Dieckmann, I. Syring, C. Ruckes, T. Nestler, H. U. Schmelz, R. Dotzauer, A. Hiester, P. Albers, D. Nettersheim, C. Bolenz, S. H. Loosen, A. Heidenreich, D. Pfister, A. Haferkamp, F. Zengerling, P. Paffenholz, GESRU Academics Testis, Penile Cancer Group, Maximilian Peter Brandt, C. Ruf, I. Syring, T. Nestler, P. Albers, D. Nettersheim, F. Zengerling, P. Paffenholz

**Affiliations:** 1 Department of Urology and Pediatric Urology, University Medical Center Mainz, University of Mainz, Langenbeckstrasse 1, 55131 Mainz, Germany; 2 Department of Urology, Bundeswehrkrankenhaus Ulm, Ulm, Germany; 3 Department of Urology, Asklepios Klinik Altona, Hamburg, Germany; 4 Klinik Und Poliklinik Für Urologie Und Kinderurologie, Universitätsklinikum Bonn, Bonn, Germany; 5 Interdisciplinary Center for Clinical Trials (IZKS), Mainz, Germany; 6 Department of Urology, Federal Armed Service Hospital Koblenz, Koblenz, Germany; 7 Department of Urology, Düsseldorf University Hospital, Düsseldorf, Germany; 8 Department of Urology, Urological Research Lab, Translational UroOncology, University Hospital Düsseldorf, Düsseldorf, Germany; 9 Department of Urology, Ulm University Hospital, Ulm, Germany; 10 Clinic for Gastroenterology, Hepatology and Infectious Diseases, University Hospital Düsseldorf, Düsseldorf, Germany; 11 Department of Urology, Urologic Oncology, Robot-Assisted and Specialized Urologic Surgery, University Hospital Cologne, Cologne, Germany

**Keywords:** Clinical stage IS, Non-seminoma, Seminoma, Tumor markers, Chemotherapy

## Abstract

**Purpose:**

Clinical stage I (CSI) testicular germ cell tumors (TGCT) represents disease confined to the testis without metastasis and CSIS is defined as persistently elevated tumor markers (TM) after orchiectomy, indicating subclinical metastatic disease. This study aims at assessing clinical characteristics and oncological outcome in CSIS.

**Methods:**

Data from five tertiary referring centers in Germany were screened. We defined correct classification of CSIS according to EAU guidelines. TM levels, treatment and relapse-free survival were assessed and differences between predefined groups (chemotherapy, correct/incorrect CSIS) were analyzed with Fisher’s exact and Chi-square test.

**Results:**

Out of 2616 TGCT patients, 43 (1.6%) were CSIS. Thereof, 27 were correctly classified (cCSIS, 1.03%) and 16 incorrectly classified (iCSIS). TMs that defined cCSIS were in 12 (44.4%), 10 (37%), 3 (11.1%) and 2 (7.4%) patients AFP, ß-HCG, AFP plus ß-HCG and LDH, respectively. In the cCSIS group, six patients were seminoma and 21 non-seminoma. Treatment consisted of active surveillance, carboplatin-mono AUC7 and BEP (bleomycin, etoposide and cisplatin). No difference between cCSIS and iCSIS with respect to applied chemotherapy was found (*p* = 0.830). 5-year relapse-free survival was 88.9% and three patients (11%) in the cCSIS group relapsed. All underwent salvage treatment (3xBEP) with no documented death.

**Conclusion:**

Around 1% of all TGCT were classified as cCSIS patients. Identification of cCSIS is of critical importance to avoid disease progression and relapses by adequate treatment. We report a high heterogeneity of treatment patterns, associated with excellent long-term survival irrespective of the initial treatment approach.

## Introduction

Testicular germ cell tumors (TGCTs) are among the most common solid tumors in men between the age of 15–35 [1, 2]. Correct clinical staging for TGCTs depends on the histologic subtype including pathohistology, radiographic evaluation of potential metastases as well as correct interpretation of serum tumor markers (TM) levels in the course of the disease [1]. 50–60% of patients initially present with increased levels of TMs (α-feto protein, AFP, β-human chorionic gonadotropin, β-HCG and lactate dehydrogenase, LDH). Clinical Stage I (CSI) is the most prevalent clinical stage in TGCT patients, which is defined as disease limited to the testis without any radiographic signs of metastases. However, there is a unique subgroup of patients in CSI with inadequately declining, persistently elevated or even increasing TMs after orchiectomy in the presence of negative cross-sectional imaging studies, which is characterized as clinical stage IS (CSIS). The clinical significance of this particular subgroup is little understood and the true prevalence is not well specified. Previous studies reported relative frequencies of < 5% of all TGCTs [3]. However, it must be assumed that a number of CSIS cases are incorrectly characterized due to misinterpretation of for example mildly elevated AFP [4, 5]. CSIS may occur in seminomas as well as in non-seminomas and all of the three classical tumor markers may be involved [6, 7]. Therefore, a wide variety of clinical features may occur in CSIs patients and little is known about the relative frequencies of the various clinical patterns and their therapeutic outcomes. The European Association of Urology Guidelines on testicular cancer recommend to treat CSIS using three cycles of BEP polychemotherapy (bleomycin, etoposide and cisplatin) [1, 2]. However, as this standard chemotherapy may involve significant long-term toxicity and as most of the patients with early stage TGCT will survive, the optimal treatment modality for this rare subgroup of patients with only minimal disease burden still needs to be defined.

In this study we aimed to analyze the true frequency of patients with CSIS in a large population of TGCT patients in respect to pre- and postoperative TM values. Furthermore, we aimed to analyze the number of patients falsely characterized as CSIS. The different treatments modalities that were applied to the patients were also analyzed and the respective therapeutic outcome was recorded.

## Patients and methods

### Definition of CSIS

In concordance with the 2020 EAU-guidelines on testicular cancer, CSIS was assumed in patients who did not have any metastatic spread upon cross sectional imaging but who presented with post orchiectomy elevated levels of any of the classic TM that did not properly return to normal, increased or did not show a marker decline according to the respective half-life kinetics [1]. In the present study, the reference limits of the participating institutions were employed to characterize CSIS patients.

To ascertain correct diagnosis of CSIS, we collected and checked data on TM levels at the time of diagnosis before and after ablative surgery up to the nadir that allowed the authors to correctly interpret the appropriate TM development. CSIS was defined for each case individually in consensus of the main authors (MPB, FZ, PP). At least three consecutive TM values for all three TMs were used to ensure correct interpretation of half-life kinetics. If TMs increased after ablative surgery, no further value of the respective TM was mandated. Cases with sufficient data that did not meet the criteria of CSIS were classified as incorrect CSIS (iCSIS). Cases with missing or incomplete data that did not allow correct classification were excluded.

### Patient selection and data collection

In this retrospective multicenter analysis, we included five tertiary referring hospitals in cooperation with the German Society of Residents in Urology Academics (GESRU Academics Testis and Penile Cancer Group). TGCT patient charts from each study site were retrospectively screened for CSIS between 1999 and 2018. We registered the total number of TGCT cases as well as the number of CSIS cases. First, data from all patients with a documented clinical stage IS from a digital data file from each participating center were extracted for further analysis. In a second step, the extracted CSIS cases were analyzed for correct or incorrect clinical staging. In order to perform correct clinical staging and to further evaluate clinical data, we registered the following parameters: patient´s age, histology of primary tumor, type of TM (AFP, bHCG, or LDH), mode of postoperative TM kinetics (no timely marker decline, increasing, undulant), the primary treatment modalities, length of follow-up (months) for each patient as well as the treatment in case of relapse. The time point of cross sectional imaging in relation to the date of orchiectomy was also verified to ensure correct clinical staging. Disease free survival and overall survival was assessed until the last known visit in the respective urologic department. Approval of the local ethics committee of cologne was obtained (No. 18-008). All data were anonymously analyzed in accordance with the local ethical standards and the declaration of Helsinki.

### Statistical analysis

All data were tabulated with a commercial data base software and descriptively analyzed. Differences between the group of correct and incorrect CSIS in respect to application of chemotherapy and TM levels were calculated with Fisher-exact test or Chi-square test as appropriate. Significance was stated as *p* ≤ 0.05. Disease-free survival was calculated with the Kaplan–Meier method.

## Results

### Patient characteristics, histopathology and tumor marker characteristics

**Fig. 1:**
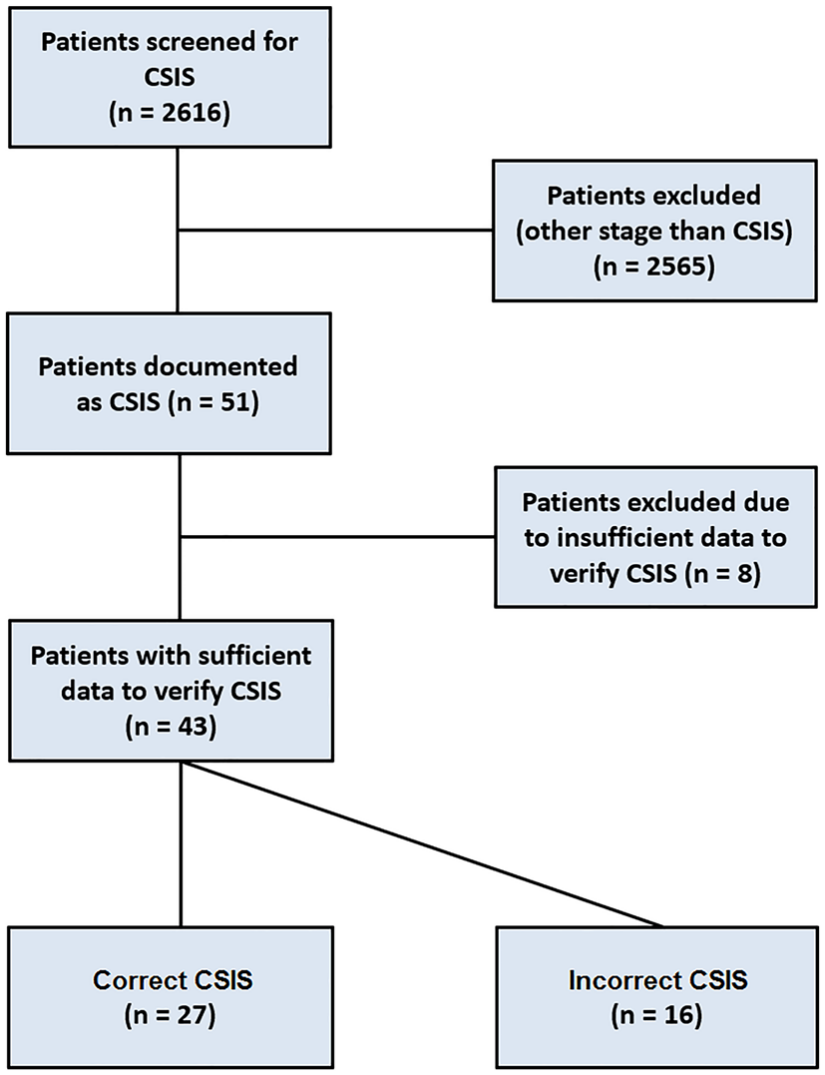
Flow chart of the screened patients

Overall, 51 patients with CSIS were identified among the 2616 TGCT patients screened. Eight patients had to be excluded due to insufficient data that did not allow to ascertain CSIS classification. The final cohort consisted of 43 patients of whom 16 revealed to be incorrectly classified CSIS patients and 27 correctly classified CSIS (Fig. 1). Regarding the total cohort (*n* = 43), the calculated incidence rate of cCSIS is 1.03% with 6 seminomas and 21 non-seminomas. In all CSIS patients cross sectional imaging was performed within 2 weeks after primary diagnosis. All of the 16 incorrectly classified CSIS patients were reclassified to CSIA or CSIB, with no reclassification to clinical stage II or III.

Further clinical characteristics, follow-up time, treatment, relapse and treatment of relapse of the 27 correctly classified CSIS patients are presented in Table 1. Median age was 31.5 years (IQR 27.25–41.25, missing *n* = 5) with a median follow-up time in the true CSIS group of 24.5 months (IQR 12.1–98). One patient with missing follow-up was excluded from the final analysis on follow-up and relapse data. TM kinetics suggesting CSIS was inadequate marker decline (33%), marker increase (44%), whereas 22% (*n* = 6) had undulant TMs. In all undulating TM cases, of which 50% (3/6 patients) were classified as seminoma, AFP was the elevated TM but did not exceed an absolute value of 20 ng/ml from at least three consecutive post-operative measurements. The TM that defined CSIS was AFP, ß-HCG, LDH and AFP plus ß-HCG in twelve, ten, two and three cases, respectively.

**Table 1:** Patient characteristics, tumor marker dynamics that defined CSIS, follow-up, location at relapse, IGCCCG risk category at relapse as well TM at relapse

Patients	Age (years)	Histology	Reason for CS1S	AFP (ng/ml) in undulant cases^a^	Follow-up time (months)	Therapy	Relapse	Time to relapse (months)	Site of metastases, prognosis and TM at relapse
1	26	NS	NTMD		62.1	2 × BEP	No		
2	30	NS	NTMD		143.9	3 × BEP	No		
3	45	NS	NTMD		106.2	2 × BEP	No		
4	43	S	NTMD		84.4	Carboplatin AUC7	Yes	11	Retrop. lymph nodes, good prognosis, no TM elevated
5	30	NS	NTMD		73.4	2 × BEP	No		
6	n.a	NS	NTMD		115.4	3 × BEP	No		
7	26	NS	NTMD		4	3 × BEP	No		
8	30	NS	NTMD		13.7	3 × BEP	No		
9	28	NS	NTMD		30.4	3 × BEP	No		
10	50	S	Increase		n.a	2 × BEP	No		
11	42	NS	Increase		4 (days)	carboplatin AUC7	No		
12	23	NS	Increase		5	3 × BEP	No		
13	25	NS	Increase		10	2 × BEP	No		
14	28	NS	Increase		2.2	2 × BEP	No		
15	n.a	NS	Increase		225.6	1 × BEP	No		
16	n.a	NS	Increase		153.7	3 × BEP	No		
17	n.a	NS	Increase		159.6	3 × BEP	No		
18	n.a	NS	Increase		92.1	3 × BEP	No		
19	27	NS	Increase		18.4	2 × BEP	No		
20	39	S	Increase		16	Carboplatin AUC7	Yes	13,8	Retrop. lymph nodes, good prognosis, LDH 680 IU/l
21	26	NS	Increase		19.9	3 × BEP	No		
22	35	NS	Undulant	9	18.3	Surveillance	Yes	7,8	Retrop. lymph nodes, good prognosis, AFP 20 ng/ml
23	42	NS	Undulant	15	38	2 × BEP	No		
24	47	S	Undulant	10	24	Surveillance	No		
25	33	NS	Undulant	11	3.7	3 × BEP	No		
26	36	S	Undulant	13.6	21.2	Surveillance	No		
27	36	S	Undulant	9.1	10.3	Surveillance	No		

*TM* tumor marker, *S* seminoma, *NS* non-seminoma, *CSIS* clinical stage IS, *NTMD* no timely marker decline, *BEP* bleomycin, etoposide, cisplatin

^a^Last documented post-operative measurement

### Treatment, relapse and relapse-free survival in CSIS

In the correctly classified CSIS group, surveillance was the initial treatment in four cases. BEP chemotherapy was applied in 20 cases (one cycle *n* = 1; two cycles *n* = 8, *n* = 11 with three cycles) and carboplatin AUC7 chemotherapy in two patients. One patient was recommended one cycle of carboplatin-mono AUC7 but did not receive the recommended therapy. In the incorrect CSIS group two patients were managed with surveillance, ten patients with BEP (one cycle *n* = 2, two cycles *n* = 3, *n* = 5 with three cycles) and three patients received carboplatin-mono AUC7. One patient received two cycles of PEI (cisplatin, etoposide and ifosfamide). The frequency of chemotherapy application was not different among the correctly and incorrectly classified cases with CSIS (*p* = 0.830).

**Fig. 2:**
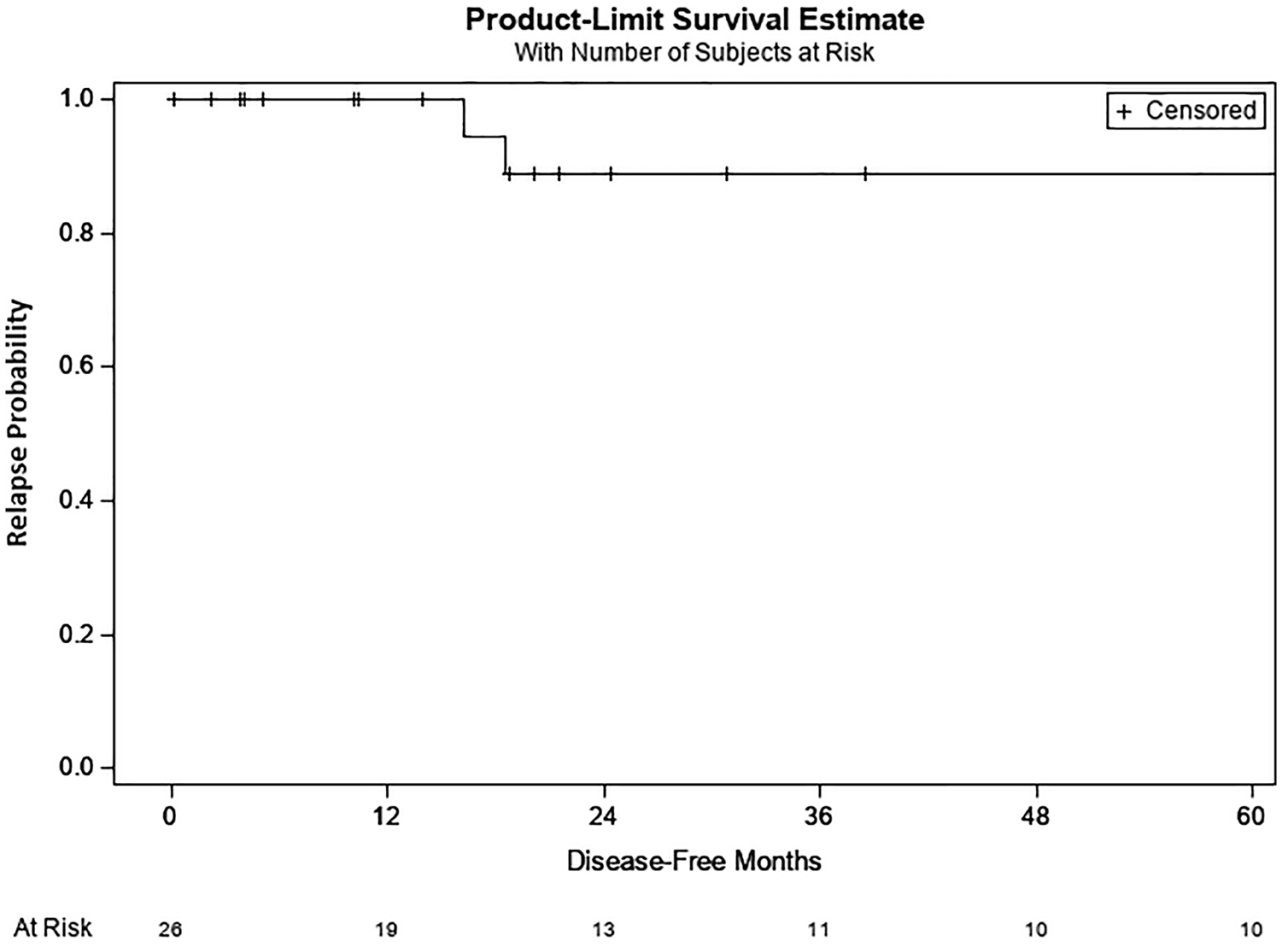
Kaplan–Meier plot of relapse probability in the correctly classified CSIS cohort

In the correctly classified CSIS group, relapse occurred in three cases (11%) after 7.8, 11 and 13.8 months one of whom initially underwent AS (non-seminoma) and two patients had initially received one cycle carboplatin-mono (both seminomas). TMs that defined CSIS in these patients were AFP for the first, β-HCG for the second and LDH for the third case of relapse. In all three cases of relapse, the classification according to IGCCCG was “good prognosis” and salvage treatment for all three patients was three cycles of BEP. In the total cCSIS cohort relapse-free survival after five and ten years was 88.9 and 77.8%, respectively (Fig. 2). No death was documented.

## Discussion

Correct clinical staging for patients with CSI TGCT, especially CSIS, is a key factor to reduce sequelae of non-guideline-conform under- or overtreatment, resulting in potential loss in treatment efficacy on the one hand and excess treatment-associated morbidity on the other hand. The incidence of documented cCSIS disease in our study was 1% for the overall cohort of 27 patients, still being lower compared to 2–5% reported in the EAU Guidelines and another recent study [1, 3]. A prior population-based study evaluated the proportion of CSIS seminoma in the SEER database and found that 28% of all clinical stage I patients were staged as CSIS. Of these LDH and β-HCG were persistently elevated in 19 and 15% of stage I seminoma patients, respectively [8]. However, only 21% of these CS1 patients had their post-orchiectomy serum tumor markers properly recorded for a correct staging as CSIS. Consequently, the authors conclude, that the number of CSIS in their study might be overestimated [8]. In our patient population, in 37% of documented CSIS, TMs returned to normal after ablative surgery and consequently these patients were incorrectly classified as CSIS. One potential explanation for misclassified CSIS might be attributed to overhasty staging and the use of pre-operative TM for final classification. Inaccurate clinical staging due to misinterpretation of TMs has also been reported by Farber and collaborators who identified incorrectly documented TM levels in up to 71% of patients with testicular cancer [4]. As a result, adjusting for overall incidence with only the correct CSIS cases in our study, incidence of CSIS was at approximately 1%. On the other hand, this approximation is limited by a possible underestimation due to correct CSIS cases that were not identified and included in our analysis due to the absence of postoperative tumor marker determinations. These circumstances highlight the urgent need to emphasize the correct classification of the TCGT patients with a focus on the correct evaluation of tumor markers according to their expected half-life kinetics after ablative surgery.

Although, guidelines recommend the use of all three tumor markers AFP, ß-HCG and LDH, they should be used with caution due to their partly non-specificity for testicular cancer. LDH, being the most unspecific TM, was found not to be a useful marker for relapse in patients on surveillance for CSI [9]. LDH can be elevated in various conditions like liver or kidney disease, myocardial infarction, hemolysis or strong exercise [10–12]. In our study, we observed two patients with elevated LDH that resulted in CSIS staging with one patient experiencing relapse. However, this finding might be due to the low case number and the retrospective design of this study. In a recent study, LDH remained elevated in 30.5–34.1%, but in concordance with our results, the colleagues point out the low specificity of LDH and state that its usefulness in clinical management is debatable [13]. On the contrary, recent data suggest that the serum level of LDH might just as well be a “new” prognostic marker in seminoma and non-seminoma according to preliminary data of the recently reported new IGCCCG-update program. Still, using LDH alone to classify a patient as CSIS might be too unspecific and must be interpreted carefully in the context of the patients’ clinical situation. According to the current IGCCCG risk classification, it is recommended, to use all three TMs LDH, AFP and ß-HCG, determining the prognosis and therapy outcome of patients with TGCT [1, 13]. In our cCSIS group, patients with increasing TM values or no timely marker decline received BEP in the majority of cases (89 and 83%, respectively) which can be considered a reasonable choice in order to achieve long term relapse free survival. On the contrary, the group with undulant AFP values, 33% of patients received BEP potentially indicating overtreatment for these patients. In another recent analysis, Wymer and coworkers evaluated the treatment pattern of 10 patients with AFP levels above normal and below 30 ng/ml, which persisted for at least 6 months without any further clinical or radiographic evidence metastasis [5]. Three of these patients received chemotherapy or retroperitoneal lymph node dissection (RPLND) based on their elevated AFP (9, 15.8 and 8.6 ng/ml), however post-treatment AFP was unchanged. Consequently, the colleagues recommend surveillance for CSIS patients with only mildly elevated and stable AFP to reduce unnecessary treatment morbidity. In our study, undulant AFP values were present in 6 patients and did not exceed 20 ng/ml. Thereof, 4 patients were treated with AS resulting in one reported relapse after 7.8 months. The remaining two patients did not experience relapse. In a recent case series with five seminoma patients and elevated AFP-values also below 20 ng/ml, AFP remained unchanged after chemotherapy treatment [14]. As a result, our data support the need of correct TM interpretation after ablative surgery, keeping in mind that AFP might be elevated due to other pre-existing medical conditions [5, 15]. Considering the drawbacks with standard TMs in clinical routine, ongoing trials are evaluating novel TMs such as the mRNA371-a-3p which showed promising results with higher sensitivity and specificity for patients experiencing relapse compared to AFP, ß-HCG and LDH [16]. Noteworthy, the TM that defined CSIS for one patient who was treated with AS had an undulant AFP value and the other patients who received carboplatin-mono AUC7 had no timely marker decline of ß-HCG and a TM increase (LDH).

Overall, three patients (11%) in the correctly classified CSIS group experienced relapse of which two were seminomas and one was non-seminoma. One patient was initially treated with AS and two patients received carboplatin-mono AUC7. Salvage treatment in our study resulted in 100% cancer specific survival which is in line with the excellent cure rates reported for CS I TGCT disease [17]. In contrast to this, no relapse occurred in the BEP treated population. Treatment patterns in our study were highly heterogeneous for CSIS patients consisting of AS, different cycles of BEP and lastly carboplatin-mono AUC7 which underlines the complexity of CSIS for treating physicians. The ideal treatment for correct CSIS is still matter of debate and to best of our knowledge there is no data available that compared the effectiveness of different treatment patterns for CSIS such as surveillance, carboplatin mono, radiotherapy and chemotherapy. However, three cycles of BEP chemotherapy is possibly the most established treatment. Interestingly, we found no difference in the relapse rate between 2 × BEP and 3 × BEP treated CSIS patients within our cohort. This might generate the hypothesis, that a lower number of PEB cycles might be sufficient to cure CSIS patients, similarly to the situation in CSI non-seminoma [18]. As our case numbers are quite low (2 × BEP: *n* = 8; 3 × BEP: *n* = 11), this remains largely hypothetical and should not be considered as standard clinical practice.

Surveillance is not regarded as a treatment option in CSIS patients according to EAU guidelines so far but has been added to the NCCN guidelines for CSIS seminoma patients [1, 19]. In line with NCCN guidelines, our data support surveillance since three out of four patients in the AS cohort did not experience relapse of which all three patients were seminomas. However, these patients were all documented as CSIS in their patient files and according to our methodology were classified as cCSIS. These cases are potentially those that would need critical discrimination whether the AFP value is elevated due to occult disease or other benign reasons. In the latter case, these patients should not have been categorized as CSIS in their patient records. Conversely, treatment with one cycle of carboplatin-mono AUC7 was not very effective and resulted in two subsequent relapses in which TMs increased or had no timely marker decline. In consequence, our data support the hypothesis that surveillance might be the adequate treatment of choice for seminoma CSIS patients with mildly elevated AFP since 75% of patients with undulant AFP values did not experience relapse and had most likely elevated AFP due to other non-malignant reasons (Table 1). Prior studies not only emphasize the potential presence of retroperitoneal disease in CSIS but also of occult metastasis, leading to the recommendation of thee cycles of BEP [20, 21]. Cisplatin-based chemotherapy leads to a high cure rate in CSIS patients with a 5 and 10-year disease-free survival rates of 87 and 85%, respectively [22]. In our study, the 5- and 10-year relapse free survival was 88.9 and 77.8%, respectively. Despite missing prospective data in CSIS, the oncologic outcome of RPLND is currently evaluated in prospective trials for CSI seminoma (NCT02797626, NCT02537548) and is considered an option in selected high risk seminoma that should be discussed as an option with the patient [1].

Radiotherapy on the other hand should not be applied in CSIS according to the current guideline recommendations. This is in line with the results of our study, as no patient was treated with radiotherapy. Additionally, a prior SEER database analysis found a decreasing number of CSIS seminoma patients treated with radiotherapy [8]. Regarding the histopathological subtype, Kamran and co-workers showed that adjuvant treatment with chemotherapy or radiation therapy for seminoma decreased over time but remained stable for non-seminoma CSIS patients who received chemotherapy or RPLND potentially indicating an increasing awareness of a risk-adapted therapy approach [3].

Our study results are hampered by a few limitations. Due to the retrospective nature of this analysis, we were unable to determine the percentage of CSI patients that received postoperative TM determinations to a sufficient extent, possibly leading to a misinterpretation of TM dynamics and underestimation of the prevalence of cCSIS patients in our cohort. Second, we did not evaluate histopathological risk factors within the overall CSIS population, for example, tumor diameter, rete testis invasion or lymphovascular invasion. In CSI seminoma and non-seminoma patients with high risk for occult disease, adjuvant treatment is a standard of care recommendation and effective in reducing the risk of relapse [1, 23, 24]. Consequently, in these cases the application of chemotherapy in CSIS is justified and should not be considered as overtreatment. In addition, radiographic disease extent was based on the respective radiological reports and we did not re-evaluate the CT or MRI scans in patients with correctly classified CSIS for reasons of availability. It might be possible that re-evaluation would have revealed lymph node involvement just above 1 cm which would in turn lead to staging as CSIIA. Finally, the median follow-up of 24.5 months is relatively short and should be considered with caution when interpreting this data.

## Conclusion

CSIS is a rare clinical situation that affected around 1% of all TGCT cases in our large patient cohort. Our study revealed incorrect classification of CSIS in around one third of these documented CSIS cases. Meticulous review of adequate post-operative TM decline, with particular caution in undulant TM course, is mandatory to identify correct CSIS, define the appropriate treatment strategy and avoid potential overtreatment in false-positive CSIS cases. BEP chemotherapy was the most common treatment applied to CSIS patients, resulting in a 100% relapse-free survival, irrespective of the number of applied chemotherapy cycles. Relapse-free survival was relatively high within our CSIS cohort and relapses were effectively treated with BEP chemotherapy. This may lead to the assumption, that not all patients with cCSIS may benefit from immediate BEP chemotherapy, especially when TM are rather slightly elevated and show no clear dynamics.

## Data Availability

Not applicable.
